# Supplementary material for: Neuronal ER-plasma membrane junctions couple excitation to Ca2+-activated PKA signaling
Source: Nat Commun. 2023 Aug 26;14:5231. doi: 10.1038/s41467-023-40930-6 (PMC10460453; doi:10.1038/s41467-023-40930-6)
Supplement: Supplementary file 1 — Supplementary Information [file 41467_2023_40930_MOESM1_ESM.pdf]

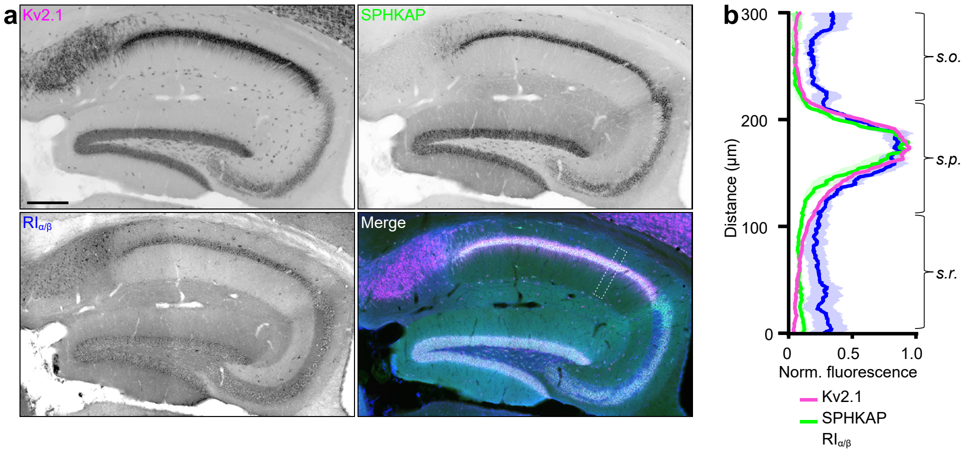

Supplementary Fig. 1. SPHKAP clusters type I PKA at Kv2.1-associated ER-PM contacts in hippocampus. a, Representative image of hippocampus in mouse brain section immunolabeled with antibodies against Kv2.1 (magenta), SPHKAP (green), and PKA-RI (blue); the white box indicates the region selected for intensity profile line scan in **b** (scale bar, 250 μm). **b**, Mean ± s.e.m. normalized immunofluorescence signal intensity of Kv2.1, SPHKAP, and RI in ROI (shown in merge panel of **a**) in hippocampal area CA1; n = 4 mice. *S.o.*, stratum oriens; *s.p.*, stratum pyramidale; *s.r.*, stratum radiatum. Source data are provided as a Source Data file.

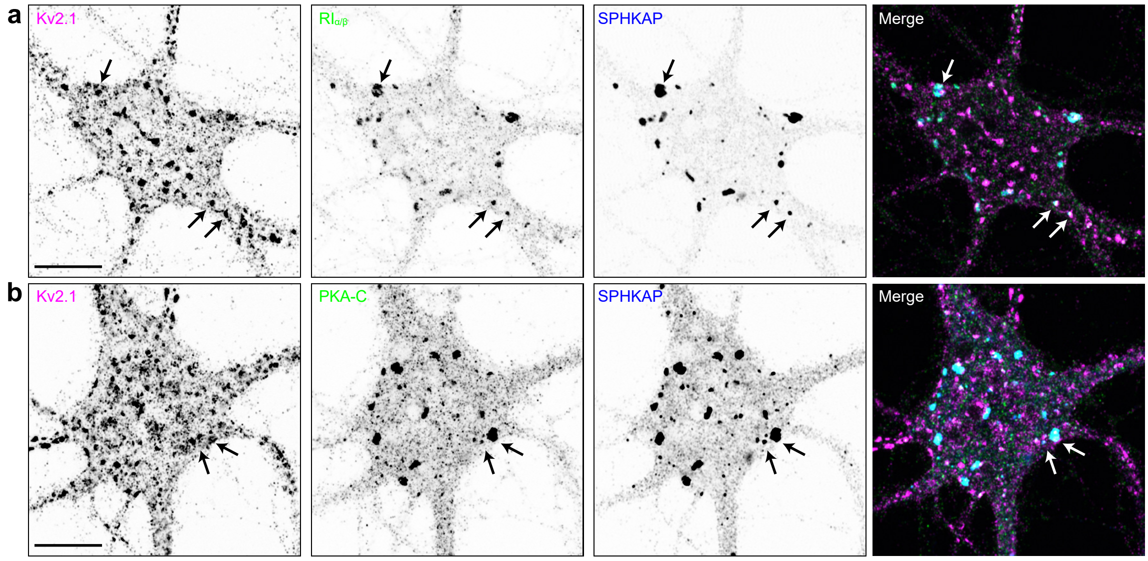

Supplementary Fig. 2. SPHKAP anchors type I PKA near Kv2.1 clusters in cultured hippocampal neurons. a-b, Representative images of hippocampal neurons (n = 3 neurons per each condition) immunolabeled for Kv2.1 (magenta), RI (green, **a**) or PKA-C (green, **b**), or SPHKAP (blue). Examples of triple co-localization are indicated by arrows (scale bar, 10 μm).

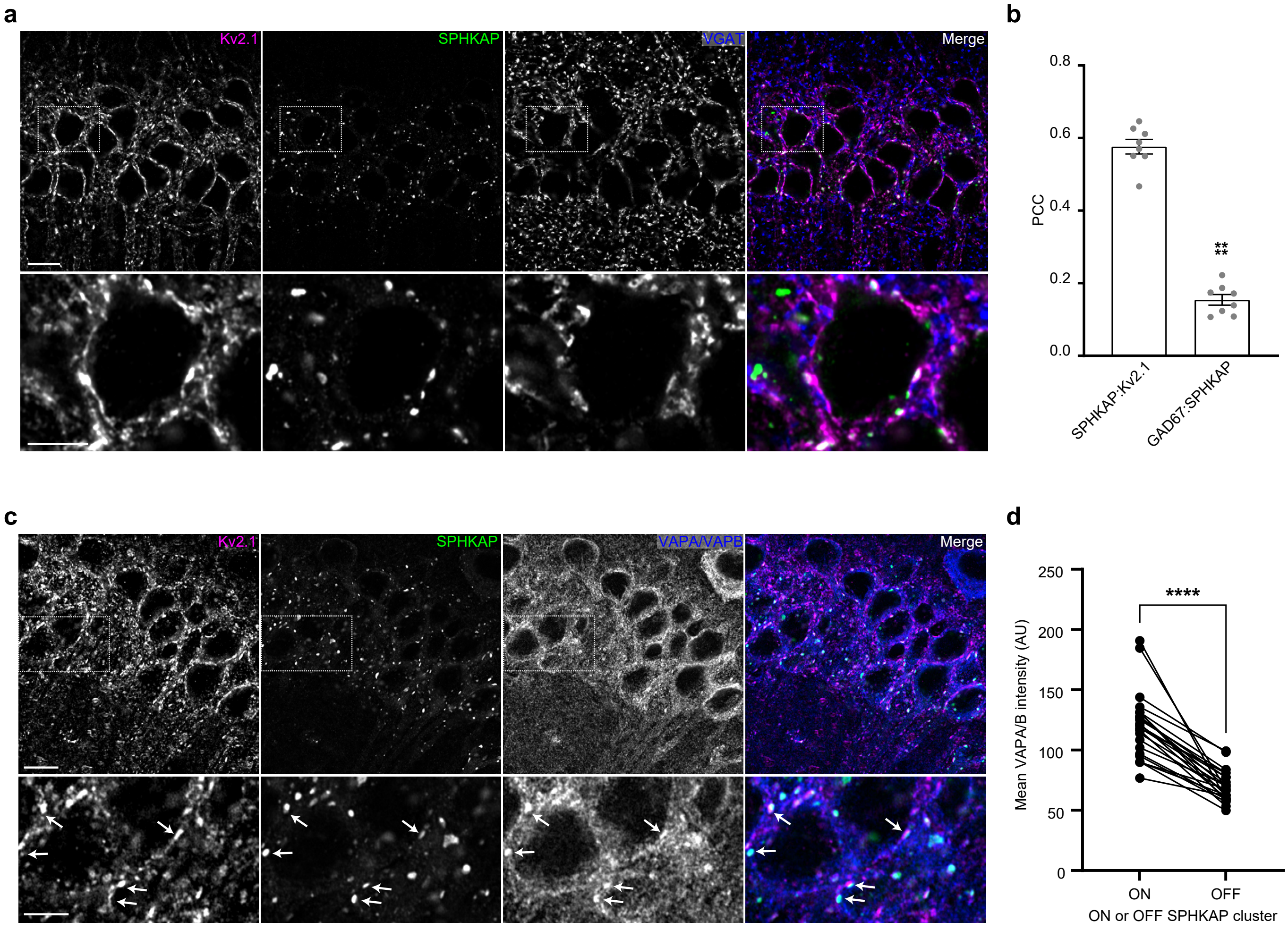

Supplementary Fig. 3. VAPA/B are enriched at sites of somatic SPHKAP clustering in hippocampal CA1 neurons. a, Stack of Zeiss AiryScan confocal images acquired from area CA1 of a mouse brain section immunolabeled for Kv2.1 (magenta), SPHKAP (green), and VGAT (blue). The row below the main panels shows expanded views of immunolabeling in the region marked by the box in the upper panels; upper scale bar, 10 µm; lower scale bar; 5 µm. Images are representative of results obtained from n = 2 mice. **b**, Colocalization (mean ± s.e.m. Pearson's colocalization coefficient, PCC) of SPHKAP with Kv2.1 or GAD67. Each point represents the mean PCC value measured from 62, 48, 63, 47, 56, 42, 49, or 62 hippocampal area CA1 somata in brain sections from 8 WT mice. Statistical significance between the mean values was determined using a two-tailed Student's *t*-test; $^{****}P < 0.0001$. **c**, As in **a**, but in a brain section immunolabeled for Kv2.1 (magenta), SPHKAP (green), and VAPA/B (blue). Examples of triple co-localization are indicated by arrows. Images are representative of results obtained from n = 2 mice. **d**, Quantification and comparison of mean VAPA/B immunofluorescence signal intensity on SPHKAP clusters (ON) or off SPHKAP clusters (OFF) measured from 24 hippocampal area CA1 somata in a mouse brain section. Statistical significance between the paired values was determined using a two-tailed Student's *t*-test; $^{****}P < 0.0001$. Source data are provided as a Source Data file.

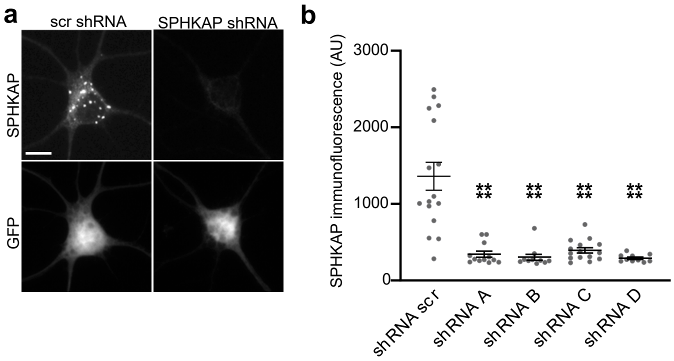

Supplementary Fig. 4. Validation of shRNA mediated SPHKAP knockdown. a, Representative images of control (scr shRNA) or SPHKAP knockdown (SPHKAP shRNA) neurons immunolabeled for SPHKAP (quantified in **b**). **b**, Mean ± s.e.m SPHKAP immunofluorescence intensity in neurons expressing indicated shRNA constructs; each point represents 1 cell (n = 16 [scr], 12 [shRNA A], 10 [shRNA B], 15 [shRNA C] and 10 [shRNA D] cells); statistical significance was determined using one-way ANOVA followed by followed by Dunnett's multiple comparisons test (vs. scr shRNA); ****$P < 0.0001$. Source data are provided as a Source Data file.

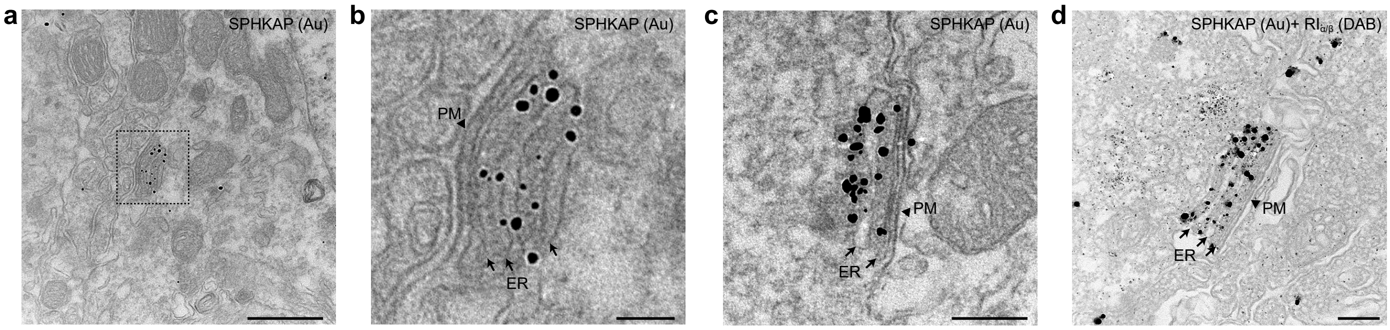

Supplementary Fig. 5. Immuno-EM reveals SPHKAP association with stacked ER cisternae in brain neurons. a-c, Representative EM images of SPHKAP-immunogold particles acquired from somata of CA1 pyramidal neurons in n = 2 mouse brain sections. Expanded view of ROI in panel **a** (box) is provided in panel **b**. Scale bars, 500 nm (**a**); 100 nm (**b**); and 200 nm (**c**). **d**, Representative EM image of SPHKAP-immunogold particles and RI-immunoperoxidase reaction product acquired from the soma of a CA1 pyramidal neuron in n = 2 mouse brain sections. Scale bar, 200 nm.

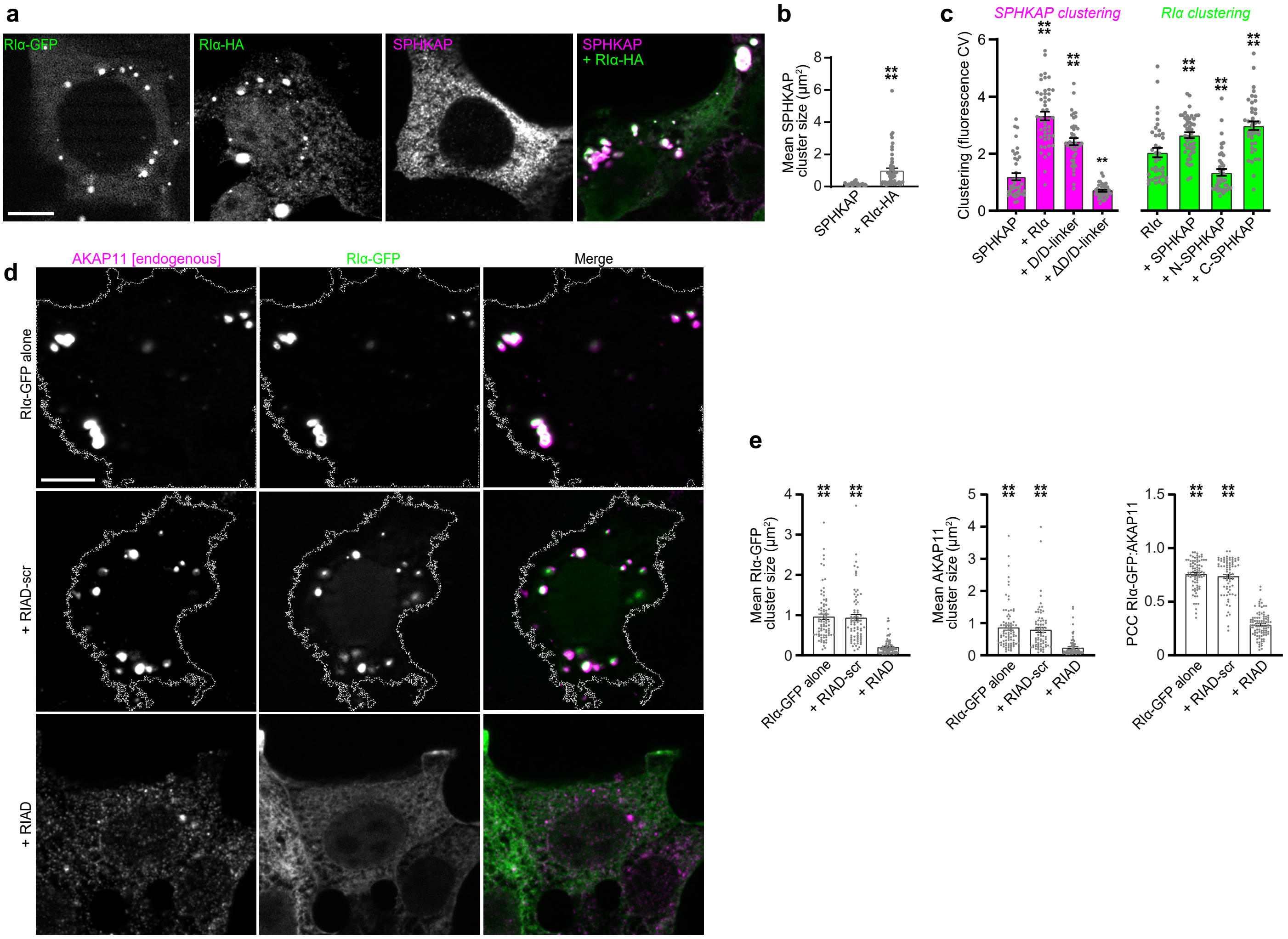

Supplementary Fig. 6. Untagged SPHKAP and HA-tagged RIα form large co-clusters and RIAD abolishes RIα-GFP-AKAP11 co-clusters in HEK cells. a, Representative images of HEK cells (quantified in **b**) expressing RIα-GFP, RIα-HA, SPHKAP, or RIα-HA and SPHKAP. Scale bar, 10 μm. **b**, Quantification of mean SPHKAP cluster size in cells expressing SPHKAP alone (n = 50 cells) or SPHKAP and RIα-HA (n = 57 cells). Statistical significance was determined using a two-tailed Mann Whitney test; $^{****}P < 0.0001$. **c**, Quantification of SPHKAP-mScarlet and RIα-GFP clustering in HEK cells. CV, coefficient of fluorescence variation. Data are mean ± s.e.m., each point represents 1 cell (n = 31 [SPHKAP], 48 [SPHKAP + RIα], 42 [SPHKAP + D/D-linker], 40 [SPHKAP + ΔD/D-linker], 39 [RIα], 32 [RIα + N-SPHKAP], and 35 [RIα + C-SPHKAP] cells). Statistical significance was determined using one-way ANOVA followed by Dunnett's multiple comparisons test (vs. SPHKAP or RI); $^{****}P < 0.0001$, $^{**}P = 0.0097$. **d**, Representative images of HEK cells transfected with RIα-GFP (green) alone or co-transfected with RIAD-scr or RIAD and immunolabeled for endogenous AKAP11 (magenta). Scale bar, 10 μm. **e**, Quantification of mean ± s.e.m. RIα-GFP cluster size (left), AKAP11 cluster size (center), or Pearson's correlation coefficient (PCC) of RIα-GFP fluorescence and AKAP11 immunofluorescence signal in 80 (RIα-GFP alone), 73 (+ RIAD-scr), and 92 (+ RIAD) cells. Statistical significance determined using one-way ANOVA followed by Tukey's multiple comparisons test (vs. RIAD); $^{****}P < 0.0001$. Source data are provided as a Source Data file.

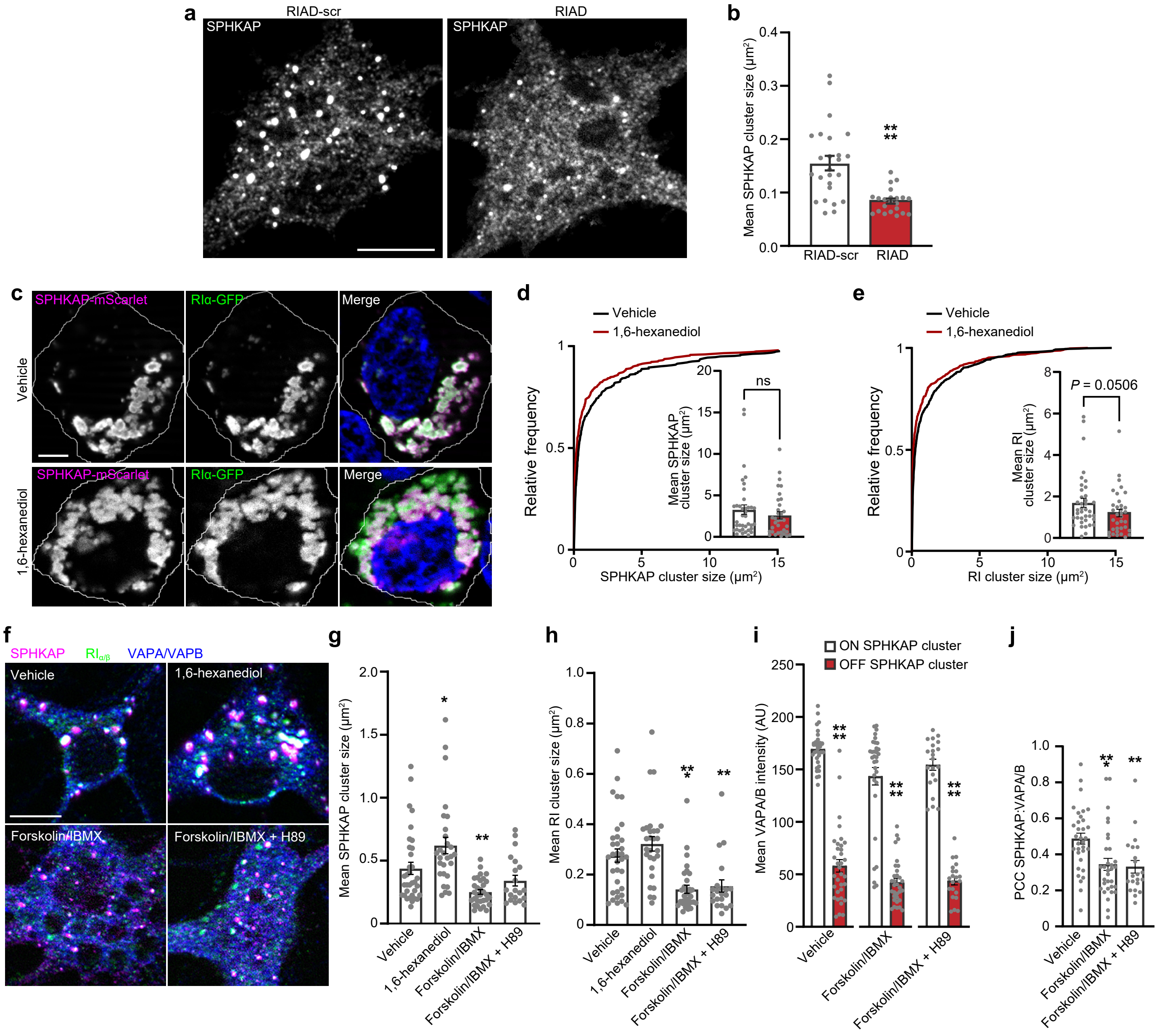

Supplementary Fig. 7. Neuronal SPHKAP and RI co-clusters are disrupted by interference with AKAP binding and by elevated cAMP, but not by 1,6-hexanediol. **a**, Representative images of neurons expressing control RIAD-scr- or RIAD-mCherry and immunolabeled for endogenous SPHKAP. Scale bar, 10 µm. **b**, Mean ± s.e.m. SPHKAP cluster area in neurons expressing RIAD-scr or RIAD; each point represents 1 cell (n = 25 [RIAD-scr] and 22 [RIAD] cells); statistical significance determined using unpaired two-tailed Student's *t*-test; ****P < 0.0001. **c**, Representative images of HEK cells co-expressing SPHKAP-mScarlet and RIα-GFP and treated with 1,6-hexanediol. Cell border indicated by white line, nuclear staining (Hoechst) is shown in blue. Scale bar, 5 µm. **d-e**, Frequency distribution and mean ± s.e.m. SPHKAP-mScarlet (**e**) or RIα-GFP (**f**) cluster area in control (Vehicle) and 1,6-hexanediol-treated HEK cells. Each point represents 1 cell (n = 38 [Vehicle] and 34 [1,6-hexanediol] cells); statistical significance assessed using unpaired two-tailed Student's *t*-test. **f**, Representative images of neurons treated with vehicle, 1,6-hexanediol, forskolin/IBMX, or forskolin/IBMX + H89 and immunolabeled for SPHKAP (magenta), RI (green), and VAPA/VAPB (blue) (scale bar, 10 µm). **g-h**, Mean ± s.e.m. somatic SPHKAP (**h**) or RI (**i**) cluster area in neurons treated with the indicated conditions. Each point represents 1 cell (n = 34 [Vehicle], 28 [1,6-hexanediol], 32 [Forskolin/IBMX], and 20 [Forskolin/IBMX + H89] cells); statistical significance determined using one-way ANOVA followed by Dunnett's multiple comparisons test (vs. vehicle); *P = 0.0147, **P = 0.0089 (**h**) or 0.0037 (**i**), ***P = 0.0002. **i**, Mean ± s.e.m. VAPA/B immunofluorescence signal intensity on SPHKAP clusters (ON) or off SPHKAP clusters (OFF) measured from 34 (Vehicle), 32 (Forskolin/IBMX), and 21 (Forskolin/IBMX + H89) treated neurons; each point represents 1 cell; Statistical significance determined using one-way ANOVA followed by Šídák's multiple comparisons test (ON vs. OFF); ****P < 0.0001. **j**, Mean ± s.e.m. Pearson's correlation coefficient values (PCC) of SPHKAP and VAPA/B immunofluorescence

measured from 33 (Vehicle), 32 (Forskolin/IBMX), and 21 (Forskolin/IBMX + H89) treated neurons; each point represents 1 cell; Statistical significance determined using one-way ANOVA followed by Tukey's multiple comparisons test (vs. vehicle); $^{***}P = 0.0046$, $^{**}P = 0.0063$. Source data are provided as a Source Data file.

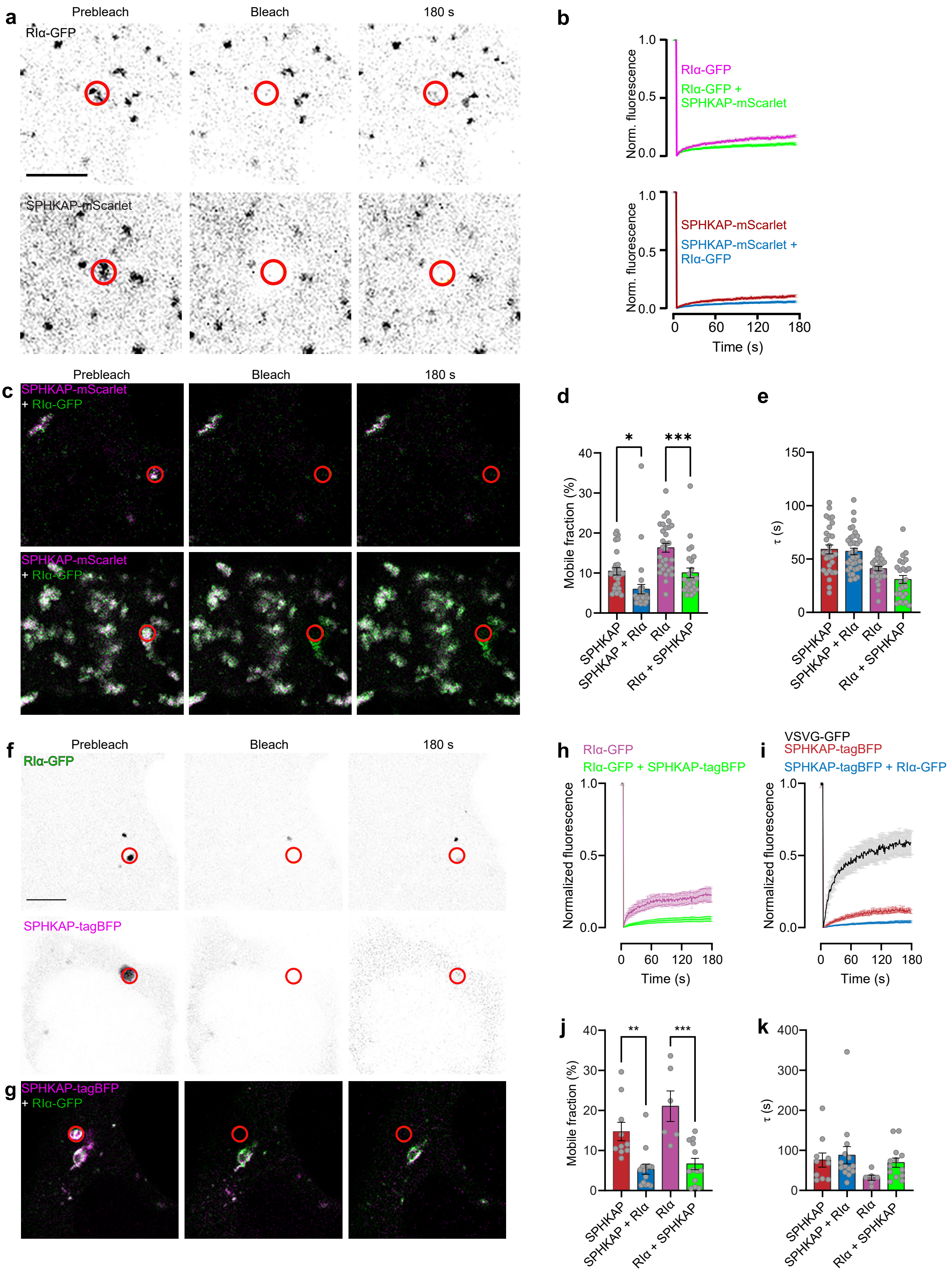

Supplementary Fig. 8. SPHKAP-RI assemblies are immobile. a, Representative images of hippocampal neurons expressing RIα-GFP (upper panels) or SPHKAP-mScarlet (lower panels) assessed by FRAP (quantified in **b**). Red circles represent areas of photobleaching. Scale bar, 5 µm. **b**, Normalized FRAP in hippocampal neurons expressing RIα-GFP (magenta) (n = 30), RIα-GFP and SPHKAP-mScarlet (green) (n = 25), SPHKAP-mScarlet (red) (n = 29), or SPHKAP-mScarlet and RIα-GFP (blue) (n = 25). Data are mean ± s.e.m. **c**, Images of FRAP in hippocampal neurons expressing RIα-GFP and SPHKAP-mScarlet (bleached ROI denoted by red circle). **d-e**, Mean ± s.e.m. mobile fraction (**d**) and recovery kinetics (**e**) of indicated constructs expressed in neurons. Each point represents a single ROI (n = 29 [SPHKAP], 30 [SPHKAP + RIα], 36 [RIα] and 25 [RIα + SPHKAP]). Statistical significance determined using one-way ANOVA followed by Tukey's multiple comparisons test; $^{***}P = 0.0010$, $^{*}P = 0.0227$. **f-g**, Representative images of FRAP in HEK cells (quantified in **h-i**) expressing RIα-GFP and SPHKAP-tagBFP separately (**f**) or together

(**g**) (bleached ROI denoted by red circle). Scale bar, 5 µm. **h**, Mean ± s.e.m. normalized FRAP curves of RIα-GFP (magenta) and RIα-GFP fluorescence when coexpressed with SPHKAP-tagBFP (green) in HEK293T cells (n = 6 [RIα] and 9 [SPHKAP + RIα]). **i**, Mean ± s.e.m. normalized FRAP of control VSVG-GFP (black), SPHKAP-tagBFP (red), or SPHKAP-tagBFP when coexpressed with RIα-GFP (blue) in HEK293T cells (n = 10 [SPHKAP], 14 [SPHKAP + RIα], and 9 [VSVG]). **j-k**, as in **d-e**, but acquired from HEK293T cells; each point represents a single ROI (n = 10 [SPHKAP], 14 [SPHKAP + RIα], 6 [RIα] and 13 [RIα + SPHKAP]). Statistical significance determined using one-way ANOVA followed by Tukey's multiple comparisons test; $^{***}P$ = 0.0002, $^{**}P$ = 0.0042. Source data are provided as a Source Data file.

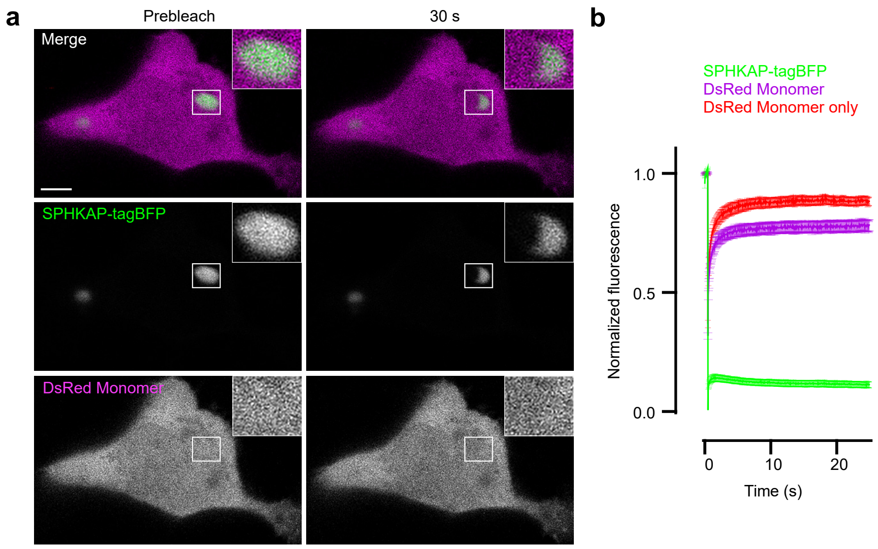

Supplementary Fig. 9. SPHKAP-RI assemblies are permeable to molecules at least as large as DsRed-monomer. a, Representative confocal images of a HEK cell co-expressing SPHKAP-tagBFP, RI-HA (not shown), and DsRed-monomer before and 30 seconds after photobleaching. Inset shows magnified view of partially photobleached SPHKAP-tagBFP cluster and DsRed fluorescence. Scale bar, 5 µm. **b**, Mean ± s.e.m. normalized FRAP curves of SPHKAP-tagBFP (green, 47 photobleached ROIs), DsRed fluorescence within a SPHKAP cluster (purple, 47 photobleached ROIs), and DsRed fluorescence in cells not expressing SPHKAP (red, 20 photobleached ROIs). Source data are provided as a Source Data file.

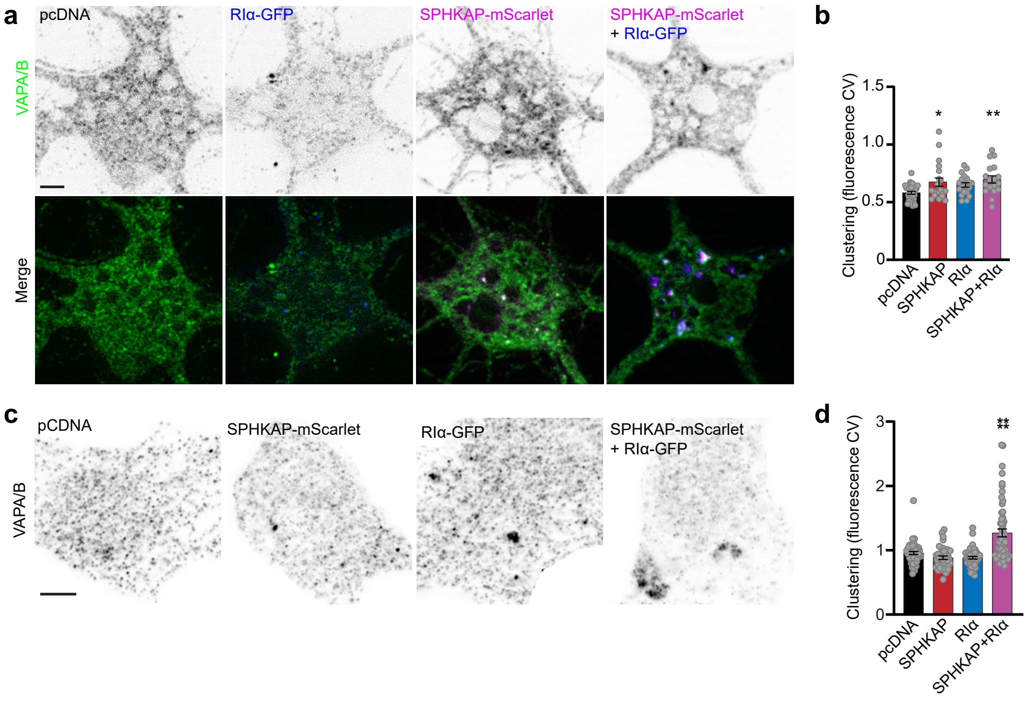

Supplementary Fig. 10. SPHKAP-RI assemblies recruit VAPs in neurons. a, Representative images of rearrangement of immunolabeled endogenous VAPA/B in cultured hippocampal neurons (**a**, quantified in **b**) and HEK cells (**c**, quantified in **d**) transfected with the indicated constructs. Scale bar, 10 µm (neurons) or 5 µm (HEK cells). **b-d**, Quantification of mean ± s.e.m. VAPA/B clustering (coefficient of variation [CV] of fluorescence intensity) in cultured hippocampal neurons (n = 33 [pcDNA], 20 [SPHKAP], 20 [RIα], and 18 [SPHKAP + RIα] cells) (**b**) and HEK cells (n = 51 [pcDNA], 39 [SPHKAP], 52 [RIα], and 57 [SPHKAP + RIα] cells) (**d**). Statistical significance was determined using one-way ANOVA followed by Tukey's multiple comparisons test (vs. pcDNA); $*P = 0.0203$, $**P = 0.0029$, $****P < 0.0001$. Each point represents 1 cell. Source data are provided as a Source Data file.

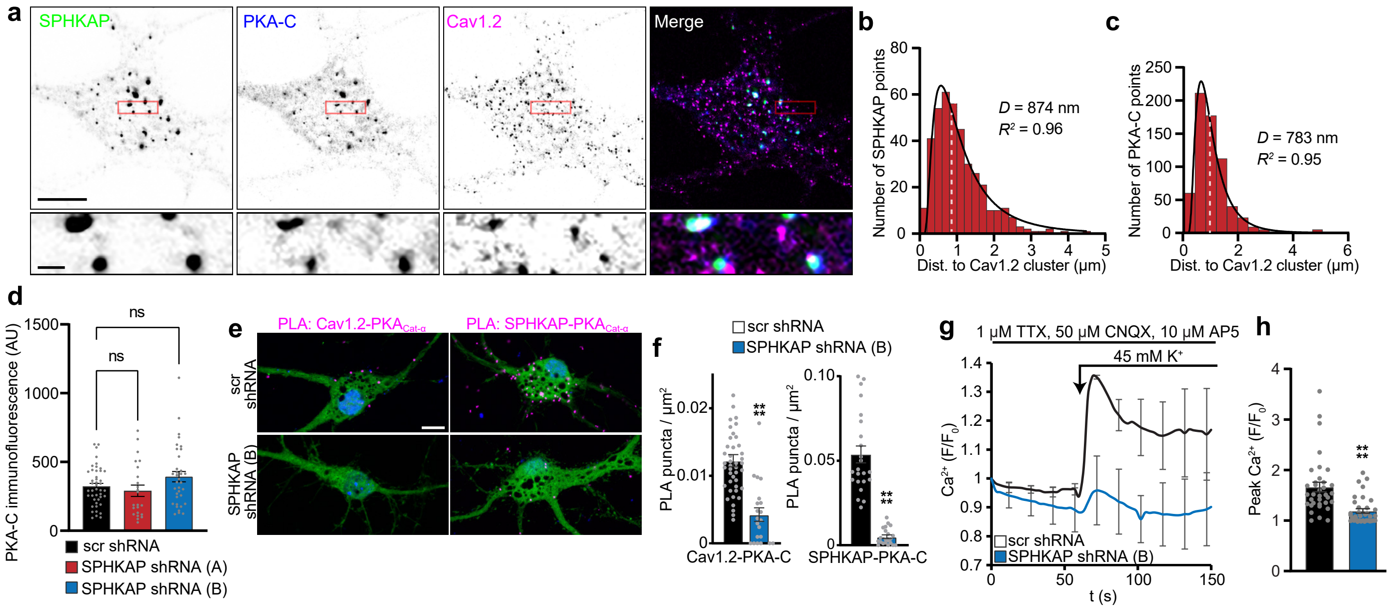

Supplementary Fig. 11. SPHKAP places PKA-C near Cav1.2 channels a, Representative images of rat hippocampal neurons (quantified in **b-c**) immunolabeled for SPHKAP (green), PKA-C (blue), and Cav1.2 (magenta). Scale bar, 10 µm. Lower, expanded views of regions marked by red boxes in upper panels. Scale bar, 1 µm. **b-c**, All-points histograms of nearest-neighbor distances of somatic SPHKAP (**c**) or PKA-C (**d**) to Cav1.2 centroids (n = 10 neurons). **d**, Mean ± s.e.m. somatic PKA-C immunofluorescence signal in 44 (scr), 23 (SPHKAP shRNA A), and 35 (SPHKAP shRNA B) neurons expressing shRNA constructs. Each point represents 1 cell; Statistical significance was determined using one-way ANOVA followed by Dunnett's multiple comparisons test (vs. scr); $P = 0.7365$ (A), $P = 0.1789$ (B). **e**, Images of control (scr shRNA) and SPHKAP KD (SPHKAP shRNA) rat hippocampal neurons subjected to proximity ligation assay (PLA) with indicated antibody pairs. shRNA-transfected cells express GFP; PLA puncta are magenta. Scale bar, 10 µm. **f**, Mean ± s.e.m. puncta density obtained in **e;** each point represents 1 cell (n = 43 [scr, Cav1.2-PKA-C], 21 [SPHKAP, Cav1.2-PKA-C], 23 [scr, SPHKAP-PKA-C], and 21 [SPHKAP, SPHKAP-PKA-C] cells). Statistical significance determined using unpaired two-tailed Student's *t*-test; ****$P < 0.0001$. **g**, Mean ± s.e.m. depolarization-induced Cal590 fluorescence in control (scr shRNA) and SPHKAP KD (SPHKAP shRNA) neurons; (n = 35 [scr] and 35 [SPHKAP] cells). **h**, Mean ± s.e.m peak Cal590 fluorescence; each point represents 1 cell (n = 35 [scr] and 35 [SPHKAP] cells). Statistical significance determined using unpaired two-tailed Student's *t*-test; ****$P < 0.0001$. Source data are provided as a Source Data file.

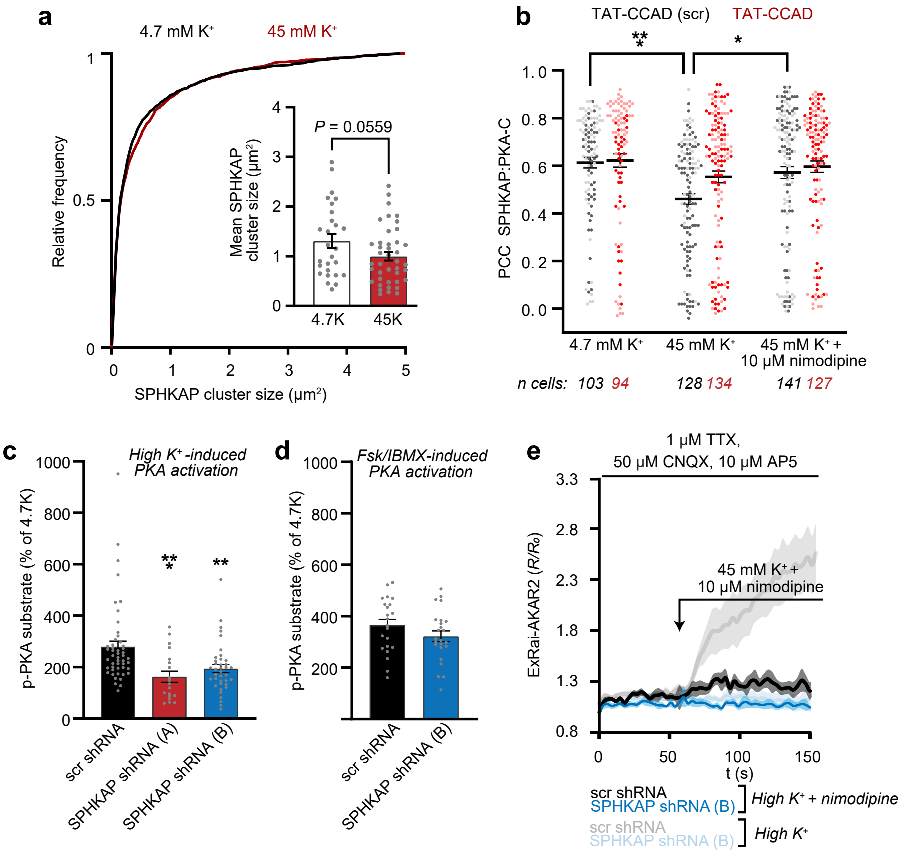

Supplementary Fig. 12. LTCC-dependent Ca^{2+} influx activates somatic PKA-C, uncoupling it from SPHKAP clusters without altering SPHKAP cluster size. **a**, Frequency distribution and mean ± s.e.m. SPHKAP cluster area in control (4.7K) and depolarized (45K) hippocampal neurons. Each point represents 1 cell (n = 27 [4.7K] and 41 [45K] cells); statistical significance was assessed using unpaired two-tailed Student's *t*-test. **b**, Mean ± s.e.m. SPHKAP:PKA-C PCC values measured in hippocampal neurons incubated overnight with TAT-CCAD-scr (control) or TAT-CCAD then stimulated with the indicated treatments. Each point represents 1 cell; shading denotes biological replicate; statistical significance was determined using one-way ANOVA followed by followed by Tukey's multiple comparisons test; ***P = 0.0004, *P = 0.0145. **c,** Mean ± s.e.m. normalized (percent of basal [4.7K]) depolarization-induced p-PKA substrate immunofluorescence signal in 49 (scr), 19 (SPHKAP shRNA A), and 38 (SPHKAP shRNA B) neurons expressing shRNA constructs. Each point represents 1 cell; statistical significance was determined using one-way ANOVA followed by Dunnett's multiple comparisons test (vs. scr); ***P = 0.0018 (A), **P = 0.0049 (B). **d**, Mean ± s.e.m. normalized (percent of basal [4.7K]) forskolin/IBMX-induced p-PKA substrate immunofluorescence signal in 22 (scr) and 24 (SPHKAP shRNA B) neurons expressing shRNA constructs. Each point represents 1 cell; statistical significance was assessed using unpaired two-tailed Student's *t*-test; P = 0.1632. **e**, Mean ± s.e.m. depolarization-induced ExRai-AKAR2 fluorescence over time in hippocampal neurons expressing control scr or SPHKAP shRNA (n = 7 [scr], and 5 [SPHKAP] cells). Source data are provided as a Source Data file.

Supplemental Table 1. Antibody information

Primary Antibodies

Antigen and antibody name	Immunogen	Manufacturer information	Validation [abbreviations: IF (immunofluorescence), IB (immunoblot), IHC (immunohistochemistry), immunoprecipitation (IP), KD (knock down), KO (knock out)]	Concentration used	Figures
Kv2.1 (KC)	Synthetic peptide aa 837-853 of rat Kv2.1 (GenBank accession number: NP_037318.1)	Rabbit pAb, in-house (Trimmer laboratory), RRID: AB_2315767	Passed IF, IB, IHC, IP, KO	Affinity purified, 1:100	Table 1 (IPs)
Kv1.2 (Kv1.2C)	Synthetic peptide aa 463-480 of rat Kv1.2	Rabbit pAb, in-house (Trimmer Laboratory), RRID:AB_2756300	Passed IF, IB, IHC, IP, KO	Affinity-purified, 1:100	Table S2 (IPs)
AMIGO-1 (L86A/37)	Fusion protein aa395-493 (cytoplasmic C-terminus) of mouse AMIGO-1 (GenBank accession number: Q80ZD8)	Mouse IgG2b mAb, In-house (Trimmer Laboratory), RRID: AB_2877364	Passed IF, IB, IHC, KO	Tissue culture supernatant, 1:5	1a, 1d
SPHKAP (L131/17)	Recombinant protein fragment corresponding to the C-terminal 126 amino acids (aa 1533-1658) of mouse SPHKAP (Uniprot accession number: E9PUC4)	Mouse IgG1 mAb, In-house (Trimmer Laboratory), RRID: AB_2877134	Passed IF, IB, IHC, IP, KD	Purified, 1 µg/mL	1a-d, 1f, 2c, 2e, 3a-c, 4f, 5e, 8a, S1, S2, S3, S4, S5, S6a, S7a,f, S11a, S11e, S12b
SPHKAP (36690)	Recombinant protein fragment corresponding to the C-terminal 126 amino acids (aa 1533-1658) of mouse SPHKAP (Uniprot accession number: E9PUC4)	Rabbit pAb, in-house (Trimmer laboratory), RRID: AB_2877133	Passed IF, IB, IHC, IP, KD	Affinity purified, 1:100	2a, 2g, 2h, 6a
PKA RI-α/β	Synthetic peptide corresponding to residues surrounding Asn310 of human PKA RI-α	Rabbit pAb, Cell Signaling Technology Cat# 3927, RRID:AB_165821	Passed IF, IB	Affinity purified, 1:100	1b, 1f, 2c, 3c, 6b, S1, S2a, S5d, S7f
PKA C-α	Synthetic peptide corresponding to the carboxy terminal sequence of human PKA C-α	Rabbit pAb, Cell Signaling Technology Cat# 4782, RRID:AB_2170170	Passed IF, IB, IP	Affinity purified, 1:100	5b, 8a, S2b, S11a, S11e, S12b
Phospho-(Ser/Thr) PKA Substrate	Synthetic phospho-PKA substrate peptide	Rabbit pAb, Cell Signaling Technology Cat# 9621, RRID:AB_330304	Passed IF, IB, IP	Affinity purified, 1:1000	8a, S12c-d
VAPA/VAPB (N479/107)	Fusion protein aa 1-219 of rat VAPA (GenBank accession number: Q9Z270)	Mouse IgG2b mAb, In-house (Trimmer laboratory), RRID:AB_2722711	Passed IF, IB, IHC, KO	Tissue culture supernatant, 2 µg/mL	2e, 2h, 5b, 5e, S3c, S7f, S10a,c
Cav1.2 (N263/31)	Fusion protein aa 808-874 (cytoplasmic loop between repeat II and III) of rat Cav1.2 (GenBank accession number: P22002)	Mouse IgG2b mAb, NeuroMab, RRID:AB_11001554	Passed IF, IB, IHC	Tissue culture supernatant, 1:2	6a, S11a,e
RyRs (34C)	Partially purified chicken pectoral muscle ryanodine receptor	Mouse IgG1 mAb, Developmental Studies Hybridoma Bank RRID: AB_528457	Passed IF, IB, IHC, IP	Concentrated tissue culture supernatant, 5 µg/mL	6a,b

Kv2.1 (K89/34)	Synthetic peptide aa 837-853 of rat Kv2.1 (GenBank accession number: NP_037318.1)	Mouse IgG1 mAb, In-house (Trimmer Laboratory), RRID: AB_2877280	Passed IF, IB, IHC, IP, KO	Purified, 2 μg/mL	2a
Kv2.1 (K89/34R)	Synthetic peptide aa 837-853 of rat Kv2.1 (GenBank accession number: NP_037318.1)	Recombinant mouse IgG2a mAb, In-house (Trimmer Laboratory), RRID: AB_2750677	Passed IF, IB, IHC, IP, KO	Tissue culture supernatant, 2 μg/mL	1b, 1f, 2g, S1a, S2, S3a
Kv2.1 (D3/71R)	Fusion protein aa 506-533 of rat Kv2.1 (GenBank accession number: NP_037318.1)	Recombinant mouse IgG2a mAb, In-house (Trimmer Laboratory), RRID: AB_2750651	Passed IF, IB, IHC, IP, KO	Tissue culture supernatant (1:5)	6a
Kv2.1 (Drk1)	Synthetic peptide aa 516-533 of rat Kv2.1 (GenBank accession number: NP_037318.1)	Rabbit pAb, In-house (Trimmer Laboratory), RRID: AB_2891234	Passed IF, IB, IHC, IP, KO	Affinity purified, 1:100	S3c
Phospho-CREB (87G3)	Synthetic phosphopeptide corresponding to residues surrounding Ser133 of human CREB	Rabbit IgG mAb, Cell Signaling Technology catalog # 9198, RRID: AB_2561044	Passed IF, IB, IHC	Purified, 1:800	7b,c
c-Fos (N486/76)	Fusion protein aa 1-377 (full-length) of human FOS (GenBank accession number: P01100)	Mouse IgG1 mAb, NeuroMab, RRID: AB_2833039	Passed IF, IB, IHC	Tissue culture supernatant, 1:5	7b,c
GAD-67 (L127/8)	Fusion protein aa 1-594 (full-length) of human GAD67 (GenBank accession number: Q99259	Mouse IgG2b mAb, In-house (Trimmer Laboratory), RRID:AB_2756509	Passed IF, IB, IHC	Tissue culture supernatant, 1:2	8c, S3b
VGAT (L118/80)	Fusion protein amino acids 1-133 (cytoplasmic N-terminus) of mouse VGAT	Mouse IgG2a mAb, In-house (Trimmer Laboratory), RRID: AB_2651169	Passed IF, IB, IHC	Tissue culture supernatant, 1:10	S3a
HA (12CA5)	Amino acids 98–106 of the human influenza virus hemagglutinin protein	Mouse IgG2b mAb, In-house (Trimmer Laboratory) RRID: AB_2532070	Passed IF, IB, IHC, IP	Purified, 1 μg/mL	S6a
AKAP11	Recombinant protein fragment corresponding to aa 1650-1901 of human AKAP11	Rabbit pAb, LSBio Cat# LS-C374339	Passed IF, IB	Affinity purified, 5 μg/mL	S6d

Secondary Antibodies

**Antibody**	**Manufacturer**	**Catalog#/RRID**	**Application [abbreviations: IF (immunofluorescence), IPr (immunoperoxidase), IG (immunogold)]**	**Concentration used**
Goat anti-Mouse IgG1 Cross-Adsorbed Secondary Antibody, Alexa Fluor 488	Invitrogen	A21121; RRID:AB_2535764	IF	1:1500
Goat anti-Mouse IgG2a Cross-Adsorbed Secondary Antibody, Alexa Fluor 488	Invitrogen	A21131; RRID:AB_2535771	IF	1:1500
Goat anti-Mouse IgG2b Cross-Adsorbed Secondary Antibody, Alexa Fluor 488	Invitrogen	A21141; RRID:AB_2535778	IF	1:1500
Goat anti-Rabbit IgG (H+L) Cross-Adsorbed Secondary Antibody, Alexa Fluor 488	Invitrogen	A11008; RRID:AB_143165	IF	1:1500
Goat anti-Mouse IgG1 Cross-Adsorbed Secondary Antibody, Alexa Fluor 555	Invitrogen	A21127; RRID:AB_2535769	IF	1:1500
Goat anti-Mouse IgG2a Cross-Adsorbed Secondary Antibody, Alexa Fluor 555	Invitrogen	A21137; RRID:AB_2535776	IF	1:1500
Goat anti-Mouse IgG2b Cross-Adsorbed Secondary Antibody, Alexa Fluor 555	Invitrogen	A21147; RRID:AB_2535783	IF	1:1500

Goat anti-Rabbit IgG (H+L) Cross-Adsorbed Secondary Antibody, Alexa Fluor 555	Invitrogen	A21428; RRID:AB_2535849	IF	1:1500
Goat anti-Mouse IgG21 Cross-Adsorbed Scondary Antibody, CF568	Biotium	20248; RRID:AB_10854985	IF	1:1500
Goat anti-Mouse IgG1 Cross-Adsorbed Secondary Antibody, Alexa Fluor 647	Invitrogen	A21240; RRID:AB_2535809	IF	1:1500
Goat anti-Mouse IgG2a Cross-Adsorbed Secondary Antibody, Alexa Fluor 647	Invitrogen	A21241; RRID:AB_2535810	IF	1:1500
Goat anti-Mouse IgG2b Cross-Adsorbed Secondary Antibody, Alexa Fluor 647	Invitrogen	A21242; RRID:AB_2535811	IF	1:1500
Goat anti-Rabbit IgG (H+L) Cross-Adsorbed Secondary Antibody, Alexa Fluor 647	Invitrogen	A21244; RRID:AB_2535812	IF	1:1500
Goat Anti-Mouse IgG2b Cross-Adsorbed Secondary Antibody, CF750	Biotium	20430	IF	1:1500
Donkey Anti-Rabbit IgG (H+L) Cross-Adsorbed Secondary Antibody, CF750	Biotium	20298; RRID:AB_10853631	IF	1:1500
Goat anti-Mouse IgG (H+L), Biotinylated	Vector Laboratories	BA-9200; RRID:AB_2336171	IPr	1:400
Goat anti-Rabbit IgG (H+L), Biotinylated	Vector Laboratories	BA-1000; RRID:AB_2313606	IPr	1:200
Goat anti-Mouse IgG (H+L), Nanogold-IgG	Nanoprobes	2001; RRID:AB_2877644	IG	1:100

Supplemental Table 2. Proteins recovered as part of Kv2.1- or Kv1.2-containing protein complexes immunopurified from DSP-crosslinked mouse brains

Kv2.1 immunopurifications

Kv2.1 K^+ channel	sp|Q03717|KCNB1_MOUSE
SPHKAP	tr|E9PUC4|E9PUC4_MOUSE
Kv2.2 K^+ channel	sp|A6H8H5|KCNB2_MOUSE
VAPA	sp|Q9WV55|VAPA_MOUSE
PKA-RI-alpha	sp|Q9DBC7|KAP0_MOUSE
PKA-RI-beta	sp|P12849|KAP1_MOUSE
Ryanodine receptor 3	sp|A2AGL3|RYR3_MOUSE (+2)
Cavβ4 Ca^{2+} channel subunit	sp|Q8R0S4|CACB4_MOUSE
VAPB	tr|Q8BH80|Q8BH80_MOUSE
Junctophilin-3	sp|Q9ET77|JPH3_MOUSE
cAMP-dependent protein kinase catalytic subunit beta	sp|P68181|KAPCB_MOUSE
Cavβ2 Ca^{2+} channel subunit	tr|C7IVS7|C7IVS7_MOUSE
Cav1.3 Ca^{2+} channel	sp|Q99246|CAC1D_MOUSE
Desmoplakin	sp|E9Q557|DESP_MOUSE
Cavβ3 Ca^{2+} channel subunit	sp|P54285|CACB3_MOUSE (+1)
Complement C5	sp|P01031|CO5_HUMAN
Hippocalcin	tr|E9PV73|E9PV73_MOUSE
Neurocalcin	tr|D3YVA2|D3YVA2_MOUSE
PKA-C alpha	sp|P05132|KAPCA_MOUSE
Sarcoplasmic/endoplasmic reticulum calcium ATPase 2	sp|O55143|AT2A2_MOUSE
Hippocalcin-like protein 1	sp|P62748|HPCL1_MOUSE
Phospholipid transfer protein C2CD2L	sp|Q80X80|C2C2L_MOUSE
Ankyrin-3	sp|G5E8K5|ANK3_MOUSE
KCC2a-S25 variant 1	tr|A0A076FR46|A0A076FR46_MOUSE
Solute carrier family 12 member 5	sp|Q91V14|S12A5_MOUSE
Cavβ1 Ca^{2+} channel subunit	tr|A0A0R4J194|A0A0R4J194_MOUSE
Methyl-CpG-binding protein 2	tr|D3Z7U4|D3Z7U4_MOUSE
Calcineurin catalytic subunit γ	sp|P48455|PP2BC_MOUSE (+1)
Ryanodine receptor 2	sp|E9Q401|RYR2_MOUSE (+1)
Calcineurin subunit B type 1	sp|Q63810|CANB1_MOUSE
Calcium-transporting ATPase	tr|E9Q828|E9Q828_MOUSE
Sarcoplasmic/endoplasmic reticulum calcium ATPase 1	sp|Q8R429|AT2A1_MOUSE
Heterogeneous nuclear ribonucleoproteins A2/B1	tr|A0A0N4SUM2|A0A0N4SUM2_MOUSE
PH and SEC7 domain-containing protein 3	sp|Q2PFD7|PSD3_MOUSE
Cav1.2 Ca^{2+} channel	tr|A0A087WSE7|A0A087WSE7_MOUSE (+3)

Kv1.2 immunopurifications

Kv1.2 K^+ channel	sp|P63141|KCNA2_MOUSE
Kv1.1 K^+ channel	sp|P16388|KCNA1_MOUSE
Kvβ2 K^+ channel	tr|Q3UPV6|Q3UPV6_MOUSE
Kv1.3 K^+ channel	sp|P16390|KCNA3_MOUSE
Kvβ1 K^+ channel	sp|P63143|KCAB1_MOUSE
Kv1.6 K^+ channel	sp|Q61923|KCNA6_MOUSE
SAP97 scaffolding protein	tr|D3Z3B8|D3Z3B8_MOUSE
Kv1.5 K^+ channel	sp|Q61762|KCNA5_MOUSE
PSD-95 scaffolding protein	sp|Q62108|DLG4_MOUSE
PSD-93 scaffolding protein	tr|E9Q2L2|E9Q2L2_MOUSE
$GABA_B$ R2	sp|Q80T41|GABR2_MOUSE
MAP7 domain-containing protein 1	sp|A2AJI0|MA7D1_MOUSE
Hsp70/GRP75	sp|P38647|GRP75_MOUSE
Mitochondrial 2-oxoglutarate/malate carrier protein	sp|Q9CR62|M2OM_MOUSE
MAGUK p55 subfamily member 3	tr|Q6XE40|Q6XE40_MOUSE
SAP102 scaffolding protein	tr|Q52KF7|Q52KF7_MOUSE
Kv1.8 K^+ channel	sp|B2RQA1|KCA10_MOUSE
Septin 8	tr|B1AQZ0|B1AQZ0_MOUSE
Myelin-oligodendrocyte glycoprotein	sp|Q61885|MOG_MOUSE (+1)
IQ motif and SEC7 domain-containing protein 1	tr|E9PUA3|E9PUA3_MOUSE
Kv1.6 K^+ channel	tr|A0A1D5RM83|A0A1D5RM83_MOUSE
