# Supplementary material for: Neuronal ER-plasma membrane junctions couple excitation to Ca2+-activated PKA signaling
Source: Nat Commun. 2023 Aug 26;14:5231. doi: 10.1038/s41467-023-40930-6 (PMC10460453; doi:10.1038/s41467-023-40930-6)
Supplement: Supplementary file 3 — Reporting Summary [file 41467_2023_40930_MOESM3_ESM.pdf]

nature portfolio

Corresponding author(s): Nicholas C. Vierra, James S. Trimmer

Last updated by author(s): Aug 14, 2023

Reporting Summary

Nature Portfolio wishes to improve the reproducibility of the work that we publish. This form provides structure for consistency and transparency in reporting. For further information on Nature Portfolio policies, see our Editorial Policies and the Editorial Policy Checklist.

Statistics

For all statistical analyses, confirm that the following items are present in the figure legend, table legend, main text, or Methods section.

n/a	Confirmed	
☐	☒	The exact sample size (*n*) for each experimental group/condition, given as a discrete number and unit of measurement
☐	☒	A statement on whether measurements were taken from distinct samples or whether the same sample was measured repeatedly
☐	☒	The statistical test(s) used AND whether they are one- or two-sided<br>*Only common tests should be described solely by name; describe more complex techniques in the Methods section.*
☐	☒	A description of all covariates tested
☐	☒	A description of any assumptions or corrections, such as tests of normality and adjustment for multiple comparisons
☐	☒	A full description of the statistical parameters including central tendency (e.g. means) or other basic estimates (e.g. regression coefficient) AND variation (e.g. standard deviation) or associated estimates of uncertainty (e.g. confidence intervals)
☐	☒	For null hypothesis testing, the test statistic (e.g. *F*, *t*, *r*) with confidence intervals, effect sizes, degrees of freedom and *P* value noted<br>*Give P values as exact values whenever suitable.*
☒	☐	For Bayesian analysis, information on the choice of priors and Markov chain Monte Carlo settings
☒	☐	For hierarchical and complex designs, identification of the appropriate level for tests and full reporting of outcomes
☐	☒	Estimates of effect sizes (e.g. Cohen's *d*, Pearson's *r*), indicating how they were calculated

Our web collection on statistics for biologists contains articles on many of the points above.

Software and code

Policy information about availability of computer code

Data collection	Zeiss Zen v2.3 SP1 [LSM880], v3.4.91 [AxioObserver]<br>Zeiss AxioVision v4.8.2.0 [AxioObserver]<br>Leica LAS X (v3.7.2.22383) [GSD microscopy]<br>ThermoFisher Proteome Discoverer v1.3 [LC-MS]
Data analysis	ImageJ v1.53q/Fiji 2.3.0 and RStudio 2022.07.1+554 [R version 4.2.1 (2022-06-23 ucrt)] were used for image analysis. X! Tandem (v Alanine (2017. 2. 1.4)) and Scaffold v4.8.4 were used for analysis of MS data. All statistical analyses were performed using GraphPad Prism v9.

For manuscripts utilizing custom algorithms or software that are central to the research but not yet described in published literature, software must be made available to editors and reviewers. We strongly encourage code deposition in a community repository (e.g. GitHub). See the Nature Portfolio guidelines for submitting code & software for further information.

Data

Policy information about availability of data

All manuscripts must include a data availability statement. This statement should provide the following information, where applicable:

- Accession codes, unique identifiers, or web links for publicly available datasets
- A description of any restrictions on data availability
- For clinical datasets or third party data, please ensure that the statement adheres to our policy

All data supporting the findings of this work can be found in the source data file and Supplementary Information; additional raw data will be made available upon request. The proteomics data generated in this study have been deposited to the ProteomeXchange Consortium via the PRIDE partner repository with the dataset identifier PXD044574. Source data are provided with this paper.

Research involving human participants, their data, or biological material

Policy information about studies with human participants or human data. See also policy information about sex, gender (identity/presentation), and sexual orientation and race, ethnicity and racism.

Reporting on sex and gender	n/a
Reporting on race, ethnicity, or other socially relevant groupings	n/a
Population characteristics	n/a
Recruitment	n/a
Ethics oversight	n/a

Note that full information on the approval of the study protocol must also be provided in the manuscript.

Field-specific reporting

Please select the one below that is the best fit for your research. If you are not sure, read the appropriate sections before making your selection.

☒ Life sciences ☐ Behavioural & social sciences ☐ Ecological, evolutionary & environmental sciences

For a reference copy of the document with all sections, see nature.com/documents/nr-reporting-summary-flat.pdf

Life sciences study design

All studies must disclose on these points even when the disclosure is negative.

Sample size	The number of replicates is based on previously published observations to reach statistical differences between datasets. See methods section for references.
Data exclusions	Cells displaying clearly unhealthy and/or unusual morphological characteristics were excluded
Replication	All experiments were successfully replicated. Experiment-by-experiment details are provided in the figure legends and/or the methods.
Randomization	Cells were randomly seeded and images were acquired by tiling large fields and all cells included in the analysis.
Blinding	The identity of samples was not known during data acquisition or analysis. Objective, quantitative data were generated and analyzed in all experiments, and no subjective classifications were performed.

Reporting for specific materials, systems and methods

We require information from authors about some types of materials, experimental systems and methods used in many studies. Here, indicate whether each material, system or method listed is relevant to your study. If you are not sure if a list item applies to your research, read the appropriate section before selecting a response.

Materials & experimental systems

n/a	Involved in the study	
☐	☒	Antibodies
☐	☒	Eukaryotic cell lines
☒	☐	Palaeontology and archaeology
☐	☒	Animals and other organisms
☒	☐	Clinical data
☒	☐	Dual use research of concern
☒	☐	Plants

Methods

n/a	Involved in the study	
☒	☐	ChIP-seq
☒	☐	Flow cytometry
☒	☐	MRI-based neuroimaging

Antibodies

Antibodies used	See Supplementary Table 1
Validation	See Supplementary Table 1

Eukaryotic cell lines

Policy information about cell lines and Sex and Gender in Research

Cell line source(s)	HEK293T cells were obtained from ATCC (CRL-3216)
Authentication	HEK293T cells were authenticated by STR profiling by the vendor.
Mycoplasma contamination	All cell lines were determined to be free of mycoplasma based on monthly ELISA for mycobacterial contamination.
Commonly misidentified lines (See ICLAC register)	No commonly misidentified cells lines were used in the study.

Animals and other research organisms

Policy information about studies involving animals; ARRIVE guidelines recommended for reporting animal research, and Sex and Gender in Research

Laboratory animals	Embryonic E18.5 Sprague-Dawley male and female rats were used to generate neuronal cultures. 8-12 week old WT, Kv2.1 KO, Kv2.2 KO, and Kv2 KO C57BL6/J male and female mice were used to generate brain sections for immunolabeling. Mice were housed in a 12-hr dark-light cycle. Mice were conventionally housed with access to food and water ad libitum.
Wild animals	The study did not involve wild animals.
Reporting on sex	Information on sex was not collected.
Field-collected samples	The study did not involve any field-collected samples.
Ethics oversight	University of California Davis IACUC, OHSU IACUC, Austrian Federal Ministry of Science and Research

Note that full information on the approval of the study protocol must also be provided in the manuscript.
